# Preference for sublingual immunotherapy with tablets in a Spanish population with allergic rhinitis

**DOI:** 10.1002/clt2.12118

**Published:** 2022-02-04

**Authors:** Mette Bøgelund, Ana Rosado Ingelmo, Jose María Ausín Ruiz, Adolfo Galán Vivó, Henrik Brandi, Mikkel Hasse Pedersen, Anne Sofie Ledgaard Loftager, Mark Aagren

**Affiliations:** ^1^ Incentive Denmark Holte Denmark; ^2^ Allergy Unit Hospital Universitario Fundación Alcorcón Spain; ^3^ Asociación Aire Libre Granada Spain; ^4^ Market Access ALK‐Abelló, S.A. Madrid Spain; ^5^ Global Market Access & Public Affairs ALK Hørsholm Denmark; ^6^ Global Pricing & Market Access LEO Pharma Ballerup Denmark

**Keywords:** allergic rhinitis, discrete choice experiment, patient preferences, subcutaneous immunotherapy, sublingual immunotherapy

## Abstract

**Background:**

This study investigated patients' preference for allergy immunotherapy (AIT) administered as either sublingual immunotherapy‐tablets versus monthly or weekly subcutaneous immunotherapy (SCIT) from a Spanish patient perspective.

**Methods:**

A discrete choice experiment (DCE) consisting of two blocks with eight choice sets in each was constructed to elicit the preferences for AIT. Three attributes were included in the DCE for the mode of administration, including the frequency of administration, the risk of systemic reactions and the co‐payment. Adults and caregivers of children with moderate to severe allergic rhinitis (AR) were included if they were not currently receiving or had not previously received AIT.

**Results:**

In total, 587 adults and 613 caregivers started the survey. Of those, 579 adults and 611 caregivers completed the survey and were included in the study. Both adults and caregivers had a significant preference for tablets compared with both monthly and weekly injections (*p* *≤* 0.0001). Furthermore, the respondents showed a significant preference for reducing the risk of systemic reactions. Subgroup analyses showed that caregivers of polyallergic children and female caregivers were significantly less price sensitive when choosing their preferred treatment.

**Conclusion:**

Our study demonstrated that both adults with AR and caregivers of children with AR prefer daily SLIT‐tablets to SCIT with either a weekly or monthly dose schedule.

## 1 INTRODUCTION

Allergic rhinitis (AR) is a non‐infectious inflammatory immunoglobulin‐E (IgE)‐mediated disease that affects the nasal mucosa in sensitised persons.^1^, ^2^ The prevalence of clinically diagnosed AR in Spanish adults has been estimated to 21.5%.^3^ AR is associated with several comorbid disorders, including conjunctivitis, atopic dermatitis and allergic asthma,^4^, ^5^ and impairs the individual's quality of life, concentration, productivity and sleep.^4^, ^6^

Allergic rhinitis can be managed by avoiding allergens, educating the patient to understand the relationship between exposure and symptoms, pharmacological treatment to relieve the symptoms and allergy immunotherapy (AIT). Nasal corticosteroids and antihistamines are recommended as first‐line therapy.^4^, ^5^ People who are sub‐optimally controlled on symptom‐relieving therapy and who have a confirmed IgE‐mediated disease are candidates for AIT.^5^, ^7^ Compared to symptom‐relieving therapy, AIT targets the underlying pathophysiology and thereby modifies the immune pathways responsible for the allergic reaction. Allergen products are not generic, but some products have demonstrated capacity to cause a long‐term disease‐modifying effect against the treated allergen(s) after cessation of therapy.^8^, ^9^, ^10^, ^11^ To attain the long‐term effect of AIT, a minimum of 3 years of therapy is recommended. AIT can be administered either as subcutaneous immunotherapy (SCIT) or sublingual immunotherapy (sublingual immunotherapy [SLIT]) as either SLIT‐tablets or SLIT‐drops in combination with pharmacological treatment. The focus of this article is restricted to SLIT‐tablets and SCIT. Subcutaneous immunotherapy is always administered by a healthcare professional in a clinical setting, whereas SLIT‐tablets can be administered at home with the first dose administered in a clinical setting. Due to the risk of severe systemic reactions, the patient should be monitored for at least 30 min after the first SLIT‐tablet or after every SCIT.^5^, ^11^ SCIT is administered using different dosing schedules usually consisting of an up‐dosing phase at increasing concentrations of allergen until reaching the maintenance dose. The length of the treatment and frequency of administration differ among products and dosing schedules.^12^ SLIT‐tablets are typically administered once daily, some with an up‐dosing period, some at the same dose throughout the whole treatment.^12^ The utilisation of SLIT‐tablet and SCIT varies across European countries. In Spain, SCIT is traditionally preferred, resulting in SLIT‐tablet being prescribed more seldomly.^13^

A systematic review and economic evaluation investigated the cost‐effectiveness of monotherapy with SLIT‐tablets and SCIT versus symptomatic therapy in the UK. Due to the cost of the AIT itself, treatment with AIT was more costly than symptomatic therapy in the first 3 years. After the first 3 years, the annual cost was higher for the patients receiving symptomatic therapy only. Compared to symptomatic therapy, SCIT and SLIT‐tablets were considered cost‐effective at year six (ICER £29,579 and £27,269, respectively) from an National Health Service and patient perspective.^14^

When deciding on a course of treatment for a patient, the healthcare professional should not only consider the cost or efficacy of the different alternatives available, but among other parameters also the patients' preferences. Including the patients' preference when deciding on a course of treatment has shown to positively impact treatment outcomes, as patient adherence improves.^15^, ^16^

Several factors such as mode of administration, efficacy, risk of adverse events and the price may affect whether patients prefer SLIT‐tablets or SCIT. In the literature, the evidence of patients' preference for either SLIT‐tablets or SCIT is lacking, and the few studies on this subject are contradictory. Chester et al.^17^ revealed that patients prefer SLIT‐tablets over SCIT (*p* < 0.0001) when asked to rank the modes of administration in a survey. Patient preference for SLIT‐tablets and SCIT was also investigated by Damm et al.^18^ in a German population using a discrete choice experiment (DCE). The results were inconsistent, as the respondents indicated that they prefer SLIT‐tables when asked directly, whereas the DCE resulted in higher preferences for SCIT. Dependency of local side effects, mode of administration as well as duration and number of clinic visits were not assumed by Damm et al.^18^ However, in clinical settings, it is more likely that patients experience these factors as dependent of each other.

To understand patients' preference for SLIT‐tablets or SCIT, further investigation is needed. This study aimed to investigate patients' preference for SLIT‐tablets or SCIT in both adults with AR and caregivers of children with AR in a Spanish setting.

## 2 METHODS

The preference for AIT was investigated using a survey. Adults with AR and caregivers of children with AR (aged 5–17) were invited to complete the online survey. The caregivers were asked to answer the survey on behalf of the child. The respondents were included if their AR was symptomatic of at least moderate severity and if they are not currently taking or have not previously tried AIT. The survey was presented to the respondents in Spanish.

The survey included questions assessing the respondent's (or the child's) type(s) of allergy, symptoms and medication use as well as sociodemographic questions. The quality of life of the adult respondents was assessed using EQ‐5D‐5L and the visual analogue scale. A DCE design was used to assess the respondents' preferences for AIT.

The survey was distributed through email panels to Spanish adults with AR and caregivers of children with AR in collaboration with Kantar/Gallup. Before starting the survey, all respondents gave informed consent. Respondents were fully anonymous, and they could leave the survey at any time. The study was conducted in accordance with the Declaration of Helsinki.

Data were collected from 27 October 2020 to 24 November 2020.

### 2.1 Discrete choice experiment (DCE)

A DCE can be used to estimate individuals' preferences for services, policies and interventions. A DCE is a stated preference method where several paired alternatives are presented to the participants, who choose the alternative that maximises their utility. Each alternative consists of a combination of attributes, which can take multiple levels.^19^, ^20^, ^21^ Attributes should include the most important health technology features, and the levels of each attribute should ideally cover all possible or hypothetical outcomes.^20^ The respondents' relative preferences can be elicited based on how they choose between these alternatives with different attribute levels.^19^, ^20^, ^21^

The DCE is a useful tool to measure patients' relative preference for different attributes of treatments and the trade‐offs that individuals are willing to make among these attributes. A great advantage of the DCE is that it provides rich data sources for decision‐making in healthcare.^22^ The attributes and their associated levels were presented to the respondents before the DCE module in the survey. The attributes and levels are shown in Table 1.

**TABLE 1:** Attributes and levels in the discrete choice experiment

Main attributes	Levels
Administration	Tablet at home every day with annual visits to an allergy clinic
	Weekly injections at an allergy clinic
	Monthly injections at an allergy clinic
Risk of a systemic reaction	No risk at all
	1 out of 200,000
	100 out of 200,000
Cost per month (co‐payment), EUR	0
	20
	70
	150

#### 2.1.1 Administration

Three levels were included in the administration attribute, each representing a mode and frequency of AIT administration: (1) tablets taken at home every day with annual visits to an allergy clinic; (2) weekly injections at an allergy clinic; and (3) monthly injections at an allergy clinic. The mode of administration, the local site reactions and the frequency of clinic visits are dependent in the sense that from SCIT you may experience injection site reactions and from SLIT you may experience itching under the tongue and not the other way around. Likewise with the frequency, you do not receive SCIT every day and not SLIT only once a month.

Therefore, these were correctly grouped into one administration attribute, and explained in detail before the DCE module. This stands in contrast to the study by Damm et al.^18^ where these attributes were assumed to be independent hence making the results inconclusive.

#### 2.1.2 Risk of systemic reactions

A low risk of a systemic reaction can occur with AIT. If not treated immediately, the reaction can become serious or even life‐threatening; therefore, the patients are monitored after the first SLIT administration and after every SCIT administration. Compared with SCIT, SLIT has a beneficial safety profile.^23^ A study by Dahl et al.^24^ reveal an approximately 60‐fold risk of systemic reactions with SCIT compared with SLIT. However, this study included a “No risk” and two levels with a 100‐fold difference (1 out of 200,000 people receiving AIT will experience a systemic reaction which is the approximate risk associated with SLIT and 100 out of 200,000 people receiving AIT will experience a systemic reaction) to ease the respondents' comprehension of the risk levels.

#### 2.1.3 Cost (co‐payment) per month

An attribute covering the price/co‐payment of AIT was included to assess patients' willingness to pay. Four levels were included in this attribute: €0, €20, €70 and €150. As is standard practise in DCE designs, these levels were set out to cover the full range of variation in the monthly price/co‐payment for AIT in Spain.

#### 2.1.4 discrete choice experiment question design

A full factorial choice design would lead to (3*3*4)^2^ = 1296 possible combinations of the attributes in the DCE module. In line with common practice, statistical efficiency was used to select the choice from these combinations. A D‐efficient design with Bayesian priors was generated using the NGENE software to improve the efficiency of data collection.

With the intention of covering a large spectrum of possible choice scenarios and not overburden the participants, two blocks were made in the survey, and the respondents were randomly assigned to one of them. Each block consisted of eight sets of questions, including two possible choice scenarios. The text introducing the DCE module and the DCE questions for both blocks is specified in the supplementary file.

### 2.2 The pilot of the study

As the survey used in this study have also been used in a similar study carried out in the US,^25^ the survey was already tested. Therefore, no pilot was carried out in Spain.

### 2.3 Exclusion and data validation

Respondents were excluded from the estimation of preference if they had finished the DCE module in less than 30 s. Data were validated prior to statistical analysis by checking answers for consistency and errors.

### 2.4 Statistical analysis

Questions prior to and after the DCE module were analysed using univariate descriptive statistics (means, medians, modes, frequencies).

The coefficients of the included attributes and levels of the DCE were determined using a conditional logit model. The probability of choosing alternative *j* from *n*_*j*_ choices in the choice scenario *i* can be described as:

$$P\left(j\right)=\frac{\text{exp}\left(X_{ij}^{\prime }\beta \right)}{\sum_{k\in C_{i}}\text{exp}\left(X_{ik}^{\prime }\beta \right)}$$

The 95% confidence interval (CI) was determined using bootstrapping, as the estimates could not be derived from the conditional logit estimates. Ten thousand replicates were preformed to estimate the CI. SAS Version 9.4 (SAS Institute Inc.,) was used for the statistical analysis.

To further investigate how preferences varied between respondent groups, we conducted subgroup analyses where we stratified the sample according to gender (male vs. female), number of allergies (mono‐vs. polyallergic), child's age (children 5–12 years vs. children 13–17 years) and whether or not respondents indicated that they wanted AIT for free.

## 3 RESULTS

### 3.1 Sample

**FIGURE 1:**
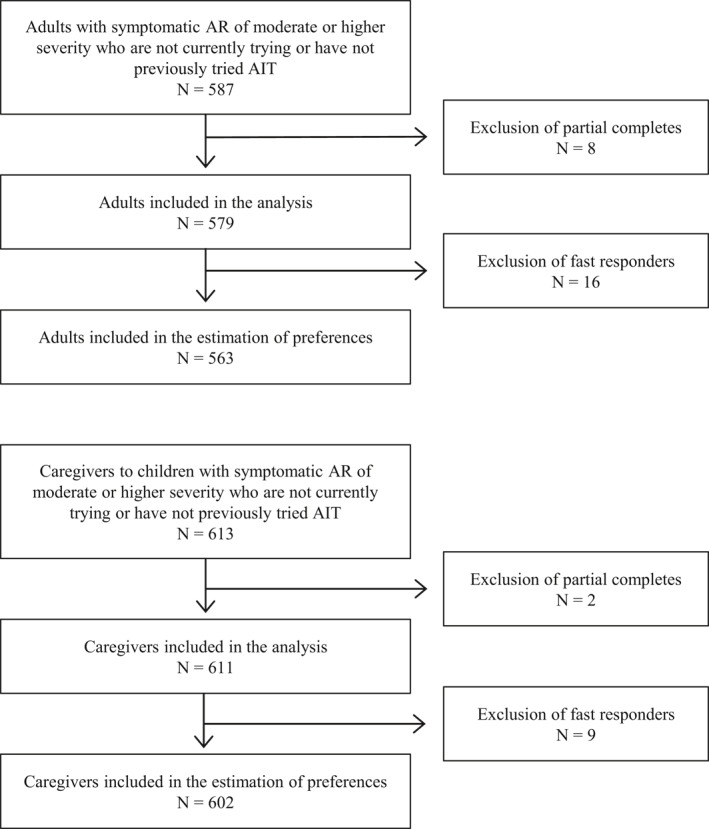
Flowchart of the study population selection

In total, 587 adults and 613 caregivers entered the survey. Of those, 8 adults and 2 caregivers did not complete the survey and were therefore excluded. This meant that 579 adults and 611 caregivers completed the survey. Of those, 16 adults and 9 caregivers answered the DCE module in less than 30 s and were therefore excluded from the preference estimates. A flowchart of the study population selection is shown in Figure 1.

Table 2 include the respondents' demographics and disease‐specific characteristics, respectively. The adults included in the study were 44 years old on average, 51% were female and their median EQ‐5D score was 0.92. The caregivers included in the survey were 42 years old on average and 57% were female. The median household income category was higher for the caregivers of children with AR compared with the adults with AR. The primary allergy for both adults with AR and children with AR was pollen, followed by house dust mites. The presence of asthma was 17% and 19% in adults with AR and children with AR, respectively. More children were polyallergic compared with adults (56% vs. 45%).

**TABLE 2:** Respondents' demographics and disease‐specific characteristics

	Adults	Caregivers answering for their children
Demographics of adults and caregivers		
Male	284 (49%)	262 (43%)
Female	295 (51%)	349 (57%)
Mean age, years	44	42
Median household income category, €	20,000–29,999	30,000–39,999
Median EQ‐5D score	0.92	
Age of allergy debut and diagnosis		
Mean age when respondent experienced first symptoms	23	6.4
Mean age of diagnosis	23	6.9
Comorbidities		
Asthma^a^	17% (99)	19% (118)
Allergies affecting the respondents the most		
Allergies towards animals (dogs, cats, horses etc.)	8% (44)	17% (101)
Allergies related to pollen (grasses, weeds, trees etc.)	48% (279)	52% (318)
Allergies related to house dust mites	36% (210)	28% (172)
Other year‐round allergies (cockroaches, mold etc.)	8% (46)	3% (20)
Allergy symptoms		
Stuffy nose, runny nose, sneezing or post‐nasal drip	88% (510)	91% (556)
Itchy, red or watery eyes	71% (411)	76% (464)
Shortness of breath, chest tightness or pain, coughing or wheezing	31% (181)	34% (208)
Itchy skin reactions, skin pain or redness of skin	33% (193)	36% (222)
Medication use		
Oral antihistamines	66% (383)	82% (498)
Decongestants	72% (268)	58% (353)
Nasal sprays and drops	35% (205)	49% (297)
Eye drops	41% (240)	42% (256)
Monoallergic and polyallergic		
Monoallergic	55% (316)	44% (266)
Polyallergic	45% (263)	56% (345)

^a^ Diagnosed by a physician (self‐reported).

### 3.2 Preferences

Adults with AR and caregivers of children with AR prefer tablets to monthly injections (*p* *≤* 0.0001). The most important attribute for both caregivers and adults was eliminating the risk of systemic reactions.

For caregivers of children with AR, we found that the preference for risk reduction was estimated to be markedly higher and the price sensitivity to be markedly lower compared with adults with AR.

The estimates from the conditional logit regression analysis are shown in Table 3. The table shows that all estimates are statistically significant and can therefore be used to predict patients' preferences when choosing AIT.

**TABLE 3:** Estimates from the conditional logit regression analysis

	Estimate	Standard error	*p*‐value
Adults with AR			
Tablets versus weekly injections	0.9194	0.0685	<0.0001
Monthly injections versus weekly injections	0.3092	0.0656	<0.0001
No risk versus risk 100/200.000	1.3482	0.0722	<0.0001
Risk 1/200,000 versus risk 100/200,000	0.7790	0.0631	<0.0001
Monthly payment in EUR	−0.0137	0.0007	<0.0001
Caregivers of children with AR			
Tablets versus weekly injections	0.8134	0.0583	<0.0001
Monthly injections versus weekly injections	0.3149	0.0582	<0.0001
No risk versus risk 100/200,000	2.2869	0.0707	<0.0001
Risk 1/200,000 versus risk 100/200,000	1.3277	0.0518	<0.0001
Monthly payment in EUR	−0.0104	0.0005	<0.0001

Abbreviation: AR, allergic rhinitis.

**FIGURE 2:**
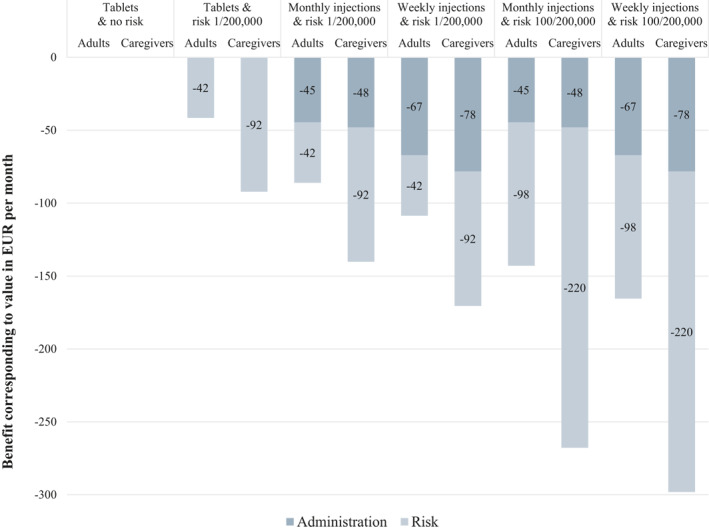
Disutility for the various attributes corresponding to value in EUR per month

Figure 2 presents for each possible product combination of attributes and levels, the estimated disutility, expressed in EUR, compared to SLIT‐tablets with no risk of systemic reactions which is indicated as respondents preferred treatment combination. The larger the disutility, the less preferred is the combination. The least preferred combination was weekly injections with a risk of systemic reactions of 100/200,000 for both adults and children.

### 3.3 Subgroup analyses

The results from the pairwise comparisons of subgroups are presented in Table 4. Overall, we found that the preferences between subgroups of respondents were similar and reflected the estimates from the general population. However, the subgroup analyses showed that caregivers of polyallergic children and female caregivers were significantly less price sensitive when choosing their preferred treatment. For adults with AR, we only detected significant differences when comparing risk preferences between male and female respondents. Specifically, we found adult female respondents with AR to have a higher preference for risk reductions than male respondents. The same was estimated for female caregivers, although this was not statistically significant.

**TABLE 4:** Results of the sub‐analyses for caregivers of children with allergic rhinitis (AR) and adults with AR, estimated preferences

Parameter estimates (standard error)	Tablets versus weekly injections	Monthly injections versus weekly injections	No risk versus risk 100/200,000	Risk 1/200,000 versus risk 100/200,000	Monthly payment in EUR
Caregivers of children aged 5–12 (*n* = 337)	0.753	0.352	2.072	1.254	−0.010
	(0.073)	(0.073)	(0.087)	(0.066)	(0.001)
Caregivers of children aged 13–17 (*n* = 265)	0.707	0.204	2.281	1.238	−0.010
	(0.085)	(0.084)	(0.102)	(0.075)	(0.001)
*p*‐value	0.659	0.909	0.067	0.558	0.28
Caregivers of monoallergic children (*n* = 261)	0.688	0.281	2.142	1.306	−0.011
	(0.083)	(0.084)	(0.100)	(0.076)	(0.001)
Caregivers of polyallergic children (*n* = 341)	0.777	0.298	2.175	1.204	−0.009
	(0.074)	(0.073)	(0.088)	(0.065)	(0.001)
*p*‐value	0.213	0.434	0.41	0.854	0.015
Male caregivers (*n* = 256)	0.736	0.271	2.084	1.266	−0.013
	(0.084)	(0.085)	(0.101)	(0.078)	(0.001)
Female caregivers (*n* = 346)	0.753	0.310	2.236	1.250	−0.008
	(0.073)	(0.073)	(0.088)	(0.065)	(0.001)
*p*‐value	0.45	0.356	0.138	0.562	<0.001
Caregivers who want AIT for free (*n* = 455)	0.690	0.241	2.173	1.238	−0.011
	(0.064)	(0.064)	(0.076)	(0.057)	(0.001)
Caregivers who do not want AIT for free (*n* = 147)	0.897	0.433	2.174	1.291	−0.008
	(0.113)	(0.112)	(0.135)	(0.099)	(0.001)
*p*‐value	0.945	0.934	0.522	0.695	0.988
Monoallergic adults (*n* = 304)	0.922	0.2694	1.3834	0.804	−0.0139
	(0.074)	(0.0712)	(0.0789)	(0.0686)	(0.000719)
Polyallergic adults (*n* = 259)	0.8152	0.3129	1.2591	0.6435	−0.0123
	(0.0787)	(0.0747)	(0.0813)	(0.0709)	(0.000705)
*p*‐value	0.1625	0.6655	0.1398	0.0536	0.9517
Males (*n* = 272)	0.864	0.2401	1.0509	0.4977	−0.0136
	(0.0767)	(0.0721)	(0.0774)	(0.0702)	(0.00072)
Females (*n* = 291)	0.8918	0.3452	1.606	0.9507	−0.0128
	(0.0768)	(0.0745)	(0.0838)	(0.0699)	(0.000709)
*p*‐value	0.5990	0.8560	<0.001	<0.001	0.7700
Adults who want AIT for free (*n* = 375)	0.8125	0.267	1.3167	0.7037	−0.0135
	(0.0657)	(0.0631)	(0.0691)	(0.0605)	(0.000623)
Adults who do not want AIT for free (*n* = 188)	0.9905	0.341	1.3375	0.7737	−0.0123
	(0.0944)	(0.0892)	(0.0985)	(0.0847)	(0.000853)
*p*‐value	0.859	0.692	0.529	0.69	0.89

Abbreviations: AIT, allergy immunotherapy; AR, allergic rhinitis.

## 4 DISCUSSION

In this study, we documented a significant preference for AIT administered as tablets compared to both weekly and monthly injections in both adults with AR and caregivers of children with AR. In addition to this, respondents showed a significant preference for reducing the risk of systemic reactions. These results confirm the findings from a similar study conducted in the US. Respondents included in the US study showed comparable preferences for AIT administered as tablets and for reductions in the risk of systemic reactions.^25^

SLIT‐tablets constitute a smaller share of the prescribed AIT in Spain compared to SCIT. More specifically, SCIT was in 2015 used for 85.5% of the adults and 77.8% of the children in Spain.^26^, ^27^ In contrast, this study documents that patients prefer SLIT‐tablets to SCIT. There is hence a gap in the current utilisation of SLIT‐tablets constituting an unmet need in AIT. Furthermore, a contributory factor to the low utilisation of SLIT‐tablets could be that only SLIT‐tablets targeting grass and dust mite allergy have marketing authorisation in Spain.^28^

As mentioned, Chester et al.^17^ and Damm et al.^18^ have earlier investigated the preference for SLIT‐tablets versus SCIT. In the study by Chester et al.^17^ the respondents were asked to rank the mode of AIT administration in a survey, whereas Damm et al.^18^ investigated the preferences using a DCE. The main finding in this study, namely that respondents prefer tablets to both monthly and weekly injections, supports the findings presented in Chester et al.^17^ where a sample of 228 adults ranked SLIT‐tablets as a preferred administration method compared to SCIT. In contrast, the results presented in this study contradict the findings presented in Damm et al.^18^ who reported inconsistencies in the respondents' preferences, as they indicated that they preferred SLIT‐tablets when asked directly, while the DCE resulted in higher preferences for SCIT. The inconsistency of the results can be explained by the authors' choice to model local side effects, mode of administration, and the duration and number of clinic visits as independent attributes.

We found that preferences for treatment only varied very modestly between relevant subgroups of adults with AR and caregivers of children with AR. We found female respondents to be more risk‐averse than male respondents, which is a general and well‐documented finding in experimental economics.^29^ Furthermore, caregivers of polyallergic children were significantly less price sensitive, this could be due to people who are polyallergic having a greater disease burden^30^ and therefore it could be hypothesised that the need for long‐term health improvements outweigh the cost of this improvement. The results presented in this study add to the existing literature on understanding patients' preferences for various attributes of AIT. Understanding patients' preferences is of the highest importance, since accounting for this when initiating patients on treatments has the potential to improve the quality of care provided and patient adherence to treatment.^16^, ^31^

Adherence to treatment is crucial for patients to obtain the clinical benefits of treatment. A large German cohort study investigating the persistence of SLIT‐tablets and SCIT (persistence defined as ≥1 prescription in both the second and third year) found that the overall proportion of persistent patients was similar in the two groups over a 3‐year period. The proportion of patients receiving SCIT who had a record of at least one prescription in year two was significantly higher than for the SLIT‐tablet population. However, the discontinuation in the third year was lower for patients receiving SLIT‐tablets compared with SCIT. This could indicate that discontinuation with SLIT‐tablets occurs earlier than for SCIT, and after 3 years, the proportions of persistent patients are similar.^32^ Similar adherence for SLIT‐tablets and SCIT is supported by a literature review, which concluded that the adherence to SLIT‐tablets and SCIT is similar.^33^

A six‐month prospective study by Sánchez^34^ investigating if patient preferences of mode of administration could improve treatment adherence during a six‐month period showed that the patients receiving their preferred route of administration were more adherent to treatment, both SLIT‐drops and SCIT, than patients for whom their physician had chosen the route of administration. There was no significant difference in adherence between those preferring SLIT‐drops and those preferring SCIT. These results emphasise the importance of including patient preference when deciding on a course of treatment. Other factors which could affect adherence are the cost of treatment/level of reimbursement and the number of follow‐ups, as demonstrated by Caruso et al.^35^ and Malet et al.^36^ respectively. As more focus is put on patient engagement in choice of therapy, conveying information on patients' preferences to healthcare providers becomes increasingly important to strengthen the basis for shared decision‐making between the patients and the healthcare provider. Published results from a survey conducted in the US showed that 72% of healthcare providers have discussed AIT options with all treatment‐eligible patients.^37^ Several shared decision‐making tools are available to assist the healthcare professional and the patient in choosing the optimal treatment.^38^, ^39^ Incorporating knowledge about patients' preferences will potentially be able to improve the precision and predictions of these tools.

The data collection for this study was carried out during the COVID‐19 pandemic. The global health situation during this period has emphasised the importance of minimising avoidable physical contact during treatment and brought increased attention to self‐administered treatment options. This may have affected the estimates of patients' preferences, thus contributing to a higher preference for tablets taken at home relative to weekly or monthly injections at the clinic. However, it is important to notice that follow‐ups cannot be completely avoided, and as mentioned above, the compliance and adherence to treatment might be affected by the number of follow‐ups received. The follow‐ups could be by telephone, which had showed high satisfaction among patients during the COVID‐19 pandemic in Spain.^40^

## 5 CONCLUSION

The findings from the present study suggest that SLIT‐tablets are preferred over SCIT among Spanish adults with AR and caregivers of children with AR. The result of this study indicates that there is a gap between clinical practice and the patients' preference for AIT in Spain. If patients' preferences are taken into consideration in the physician‐patient conversation when deciding the course of treatment, the quality of care and adherence of AIT could be improved.

## AUTHOR CONTRIBUTIONS

**Mette Bøgelund:** Conceptualization; Data curation; Investigation; Methodology; Project administration; Writing – original draft. **Ana Rosado Ingelmo:** Methodology; Validation. **Jose María Ausín Ruiz:** Methodology; Validation. **Adolfo Galán Vivó:** Conceptualization; Methodology; Resources. **Henrik Brandi:** Conceptualization; Methodology; Project administration; Supervision; Visualization; Writing – review & editing. **Mikkel Hasse Pedersen:** Conceptualization; Data curation; Formal analysis; Investigation; Methodology; Writing – original draft. **Anne Sofie Ledgaard Loftager:** Formal analysis; Writing – original draft. **Mark Aagren:** Conceptualization; Funding acquisition; Methodology; Project administration; Supervision; Visualization; Writing – review & editing.

## Supporting information

Supplementary Material
