# Supplementary material for: Preference for sublingual immunotherapy with tablets in a Spanish population with allergic rhinitis
Source: Clin Transl Allergy. 2022 Feb 4;12(2):e12118. doi: 10.1002/clt2.12118 (PMC8814907; doi:10.1002/clt2.12118)
Supplement: Supplementary file 1 — Supplementary Material [file CLT2-12-e12118-s001.docx]

Supplementary material 1

## Introduction to the DCE module

Before the DCE questions, all respondents were introduced to the attributes and levels. The following text was included in the survey:

#### Introduction

In the following section we would like to investigate the importance of various features related to treatment of allergy. The questions are hypothetical and do not refer to an actual treatment available today or in the future. Nor will the answers you provide have an effect on your future payment for medication.

Imagine that you see an allergy specialist who suggests that you get a new treatment called allergen immunotherapy (AIT) which has been approved by the Spanish Agency for Medicines and Health Products (AEMPS). The treatment will last for 3 years. The goal of AIT treatment is that your body stops overreacting to allergens and you experience both a reduction of symptoms and dependence upon symptom relieving medication.

#### Administration

The treatment is administered by the allergy specialist subsequently administered in the healthcare centre or medical centre. Please imagine that your travel time to the clinic in less than 30 minutes.

There are two ways of getting the AIT treatment:

- Injections: Treatment as subcutaneous injections in the upper arm by a nurse or a doctor at the clinic. Subcutaneous means under the skin. In this type of injection, a short thin needle is used to inject a drug into the tissue layer between the skin and the muscle in the upper arm.
- Tablets: Treatment as a rapidly dissolving non-flavoured tablet you put under the tongue.

Both treatment types are taken for 3 years and have the same effect. If you receive an AIT treatment with injections, you should visit the clinic every week to start with, then after 3-6 months you will spread your visits out to once a month.

After receiving an injection, you will be required to stay at the clinic for 30 minutes to monitor for any side effects or reactions. The injection feels like a minor pinch, and you may experience a mild local reaction at the upper arm after the injection.

If you receive an AIT treatment with tablets, you receive the first tablet at the clinic and stay at the clinic for 30 minutes to monitor for any side effects or reactions. If you do not experience any problems, you start taking the tablets at home every day. Subsequently, you should visit the clinic every 6-12 months for a check-up. In the first couple of weeks, 2 out of 3 people experience mild local reactions in the mouth or throat that last 5-10 minutes after taking the tablet.

#### Risk of systemic reaction

AIT treatment is associated with a low risk of a systemic reaction. It typically causes one or more of the following: an itchy rash, throat or tongue swelling, shortness of breath, vomiting, light-headedness, or low blood pressure. It is potentially serious or even life threatening if not treated immediately. That is why you are monitored at the clinic every time you get an injection and the first time you get a tablet.

We express the risk of getting systemic reaction like this:

- 1 out of 200,000 meaning that one out of 200,000 people receiving the AIT treatment will experience a systemic reaction.
- 10 out of 200,000 meaning that 10 out of 200,000 people receiving the AIT treatment will experience a systemic reaction.
- 100 out of 200,000 meaning that 100 out of 200,000 people receiving the AIT treatment will experience a systemic reaction.

**Cost (out or pocket per month)**

The costs shown in the following section express the net cost for you for the different treatments, taken into account the reimbursement of your medication. We call this a patient co-payment, since the remaining cost of the medication is subsidized by the state or your insurer.

#### DCE questions

In the following we will show you two different AIT options. In order to understand which of the features are most important, we would like you to choose the treatment that you would prefer.

There are no right or wrong answers. They have the exact same effect on your allergy and all other aspects of the treatment are the same.

## DCE questions

The DCE module consisted of two blocks of eight choice sets. The respondents were randomly assigned to receive one of the two blocks. An overview of the choice sets in block 1 are shown in Table S1, and the choice sets for block 2 are shown in Table S2. The choice sets shown to adults and caregivers were identical.

Table S1: The table illustrates the DCE questions in block 1.

|  |  | **Treatment A** | **Treatment B** |
| --- | --- | --- | --- |
| Question 1 | Administration | Weekly injections at an allergy clinic | Monthly injections at an allergy clinic |
|  | Risk of systemic reaction | 100 out of 200,000 | No risk at all |
|  | Cost per month (co-payment) | 20 | 70 |
| Question 2 | Administration | Tablet at home every day with annual visits to an allergy clinic | Tablet at home every day with annual visits to an allergy clinic |
|  | Risk of systemic reaction | 1 out of 200,000 | No risk at all |
|  | Cost per month (co-payment) | 0 | 150 |
| Question 3 | Administration | Tablet at home every day with annual visits to an allergy clinic | Weekly injections at an allergy clinic |
|  | Risk of systemic reaction | No risk at all | 100 out of 200,000 |
|  | Cost per month (co-payment) | 150 | 70 |
| Question 4 | Administration | Tablet at home every day with annual visits to an allergy clinic | Monthly injections at an allergy clinic |
|  | Risk of systemic reaction | No risk at all | 100 out of 200,000 |
|  | Cost per month (co-payment) | 0 | 0 |
| Question 5 | Administration | Weekly injections at an allergy clinic | Monthly injections at an allergy clinic |
|  | Risk of systemic reaction | 1 out of 200,000 | No risk at all |
|  | Cost per month (co-payment) | 70 | 20 |
| Question 6 | Administration | Monthly injections at an allergy clinic | Tablet at home every day with annual visits to an allergy clinic |
|  | Risk of systemic reaction | 100 out of 200,000 | 1 out of 200,00 |
|  | Cost per month (co-payment) | 20 | 150 |
| Question 7 | Administration | Monthly injections at an allergy clinic | Weekly injections at an allergy clinic |
|  | Risk of systemic reaction | 100 out of 200,000 | 1 out of 200,000 |
|  | Cost per month (co-payment) | 150 | 0 |
| Question 8 | Administration | Monthly injections at an allergy clinic | Tablet at home every day with annual visits to an allergy clinic |
|  | Risk of systemic reaction | 1 out of 200,000 | 100 out of 200,000 |
|  | Cost per month (co-payment) | 70 | 20 |

Table S2: The table illustrates the DCE questions in block 2.

|  |  | **Treatment A** | **Treatment B** |
| --- | --- | --- | --- |
| Question 1 | Administration | Weekly injections at an allergy clinic | Monthly injections at an allergy clinic |
|  | Risk of systemic reaction | 1 out of 200,000 | 100 out of 200,000 |
|  | Cost per month (co-payment) | 20 | 70 |
| Question 2 | Administration | Tablet at home every day with annual visits to an allergy clinic | Weekly injections at an allergy clinic |
|  | Risk of systemic reaction | 100 out of 200,000 | 1 out of 200,000 |
|  | Cost per month (co-payment) | 20 | 70 |
| Question 3 | Administration | Monthly injections at an allergy clinic | Weekly injections at an allergy clinic |
|  | Risk of systemic reaction | 100 out of 200,000 | No risk at all |
|  | Cost per month (co-payment) | 70 | 20 |
| Question 4 | Administration | Monthly injections at an allergy clinic | Weekly injections at an allergy clinic |
|  | Risk of systemic reaction | 1 out of 200,000 | 100 out of 200,000 |
|  | Cost per month (co-payment) | 70 | 20 |
| Question 5 | Administration | Tablet at home every day with annual visits to an allergy clinic | Weekly injections at an allergy clinic |
|  | Risk of systemic reaction | No risk at all | No risk at all |
|  | Cost per month (co-payment) | 0 | 150 |
| Question 6 | Administration | Weekly injections at an allergy clinic | Tablet at home every day with annual visits to an allergy clinic |
|  | Risk of systemic reaction | No risk at all | 1 out of 200,000 |
|  | Cost per month (co-payment) | 0 | 0 |
| Question 7 | Administration | Weekly injections at an allergy clinic | Monthly injections at an allergy clinic |
|  | Risk of systemic reaction | 100 out of 200,000 | 1 out of 200,000 |
|  | Cost per month (co-payment) | 150 | 0 |
| Question 8 | Administration | Weekly injections at an allergy clinic | Tablet at home every day with annual visits to an allergy clinic |
|  | Risk of systemic reaction | No risk at all | 100 out of 200,000 |
|  | Cost per month (co-payment) | 150 | 150 |
